# A socio-ecological analysis of factors influencing HIV treatment initiation and adherence among key populations in Papua New Guinea

**DOI:** 10.1186/s12889-021-12077-w

**Published:** 2021-11-04

**Authors:** Elke Mitchell, Avi Hakim, Somu Nosi, Martha Kupul, Ruthy Boli-Neo, Herick Aeno, Michelle Redman-Maclaren, Sophie Ase, Angelyn Amos, Parker Hou, Rebecca Narokobi, Barne Willie, Andrew J. Vallely, John M. Kaldor, Steven G. Badman, Angela Kelly-Hanku

**Affiliations:** 1 The Kirby Institute for Infection and Immunity in Society, UNSW Sydney, Sydney, Australia; 2 Division of Global HIV and Tuberculosis, US Centers for Disease Control and Prevention, Atlanta, GA USA; 3 Sexual and Reproductive Health Unit, Papua New Guinea Institute of Medical Research, PO Box 60, Goroka, Eastern Highlands Province Papua New Guinea; 4 College of Medicine and Dentistry, James Cook University, Cairns, Australia

**Keywords:** Papua New Guinea, HIV treatment, Key populations, Qualitative, Adherence, HIV care cascade

## Abstract

**Background:**

In Papua New Guinea (PNG) members of key populations, including female sex workers (FSW), men who have sex with men (MSM) and transgender women (TGW), have higher rates of HIV compared to the general adult population and low engagement in HIV care. This paper examines the socio-ecological factors that encourage or hinder HIV treatment initiation and adherence among HIV positive members of key populations in PNG.

**Methods:**

As part of a larger biobehavioural survey of key populations in PNG, 111 semi-structured interviews were conducted with FSW, MSM and TGW, of whom 28 identified as living with HIV. Interviews from 28 HIV positive participants are used in this analysis of the influences that enabled or inhibited HIV treatment initiation and treatment adherence.

**Results:**

Enablers included awareness of the biomedical benefits of treatment; experiences of the social, familial and health benefits of early treatment initiation and adherence; support provided by family and friends; and non-judgmental and supportive HIV service provision. Factors that inhibited treatment initiation and adherence included perception of good health and denial of HIV diagnosis; poor family support following positive diagnosis; and anonymity and stigma concerns in HIV care services.

**Conclusion:**

Exploring health promotion messages that highlight the positive health impacts of early treatment initiation and adherence; providing client-friendly services and community-based treatment initiation and supply; and rolling out HIV viral load testing across the country could improve health outcomes for these key populations.

## Background

Globally, key populations, including female sex workers (FSW), men who have sex with men (MSM) and transgender women (TGW), have increased risk of acquiring HIV and experience a greater burden of disease compared to the general adult population [1–3]. Early linkage and retention in the HIV care cascade, from testing to antiretroviral therapy (ART) initiation and adherence and HIV viral suppression, is essential to slowing disease progression in individuals and HIV transmission within the population more broadly [4, 5]. Global HIV policy supports early uptake and universal treatment for HIV [6, 7] and along with the Joint United Nations Programme on AIDS (UNAIDS) 95–95-95 targets where 95% of HIV positive people will be aware of their status; 95 of those diagnosed will receive sustained ART; and 95% of those on ART will achieve viral suppression have set the agenda of “ending AIDS” by 2030 [8]. However, key populations with HIV in low- and middle-income countries (LMICs) experience ongoing barriers to engaging with HIV services at all stages of the HIV care cascade, including treatment initiation after HIV diagnosis and low ART adherence [9–15].

In Papua New Guinea (PNG), sex work and male-to-male sex are illegal and highly stigmatised [16]. In this setting key populations including FSW, MSM and TGW are particularly vulnerable to HIV due to high prevalence of HIV-related risk behaviours, including concurrent sexual partnerships, low condom use and high prevalence of sexually transmissible infections [17, 18]. *Kauntim mi tu*, a biobehavioural survey (BBS) among FSW, MSM and TGW in PNG undertaken in 2016–17 across three cities reported an HIV prevalence of 15.2% among FSW in Port Moresby, 11.9% in Lae, and 19.6% in Mount Hagen [19]. HIV prevalence among MSM and TGW was 8.5% in Port Moresby and 6.9% in Lae [18]. These rates are significantly higher than the estimated adult prevalence of 0.9% [17]. For a detailed description of the methodology used, including reasons for combining MSM and TGW into a single survey, see the *Kauntim mi tu* study report [17].

The *PNG National STI and HIV Strategy 2018–2022* [20] calls for greater focus on key populations including community mobilisation and engagement; equitable and safe access to HIV prevention, care and support services; and peer-based outreach activities. Despite this, findings from *Kauntim mi tu* across the three sites show that PNG is falling short of reaching the UNAIDS 95–95-95 targets among these populations [16, 17]. For example, less than half of HIV positive FSW in Port Moresby (39%) self-reported awareness of their HIV status (MSM/TGW, 24.4%); of these, 79.6% self-reported taking ART (MSM/TGW, 43.9%); and of these 54.1% were virally suppressed (MSM/TGW, 86.1%) [16]. These findings indicate there is a need to better understand why key populations in PNG are not engaging in HIV care services and why they are not virally supressed [16].

While behavioural data can quantify the extent to which people are linked to care, and possible associations, for example, qualitative research on the other hand provides detailed examination of the diverse influences that affect people’s linkage and retention in HIV care [21]. In the Asia Pacific region there is limited, but important, qualitative research that explores the role of socio-ecological influences on ART initiation and adherence among FSW, [11, 22, 23] MSM, [12, 23–27] and TGW [14, 25]. One of the earliest works on socio-ecological models in health was written by McLeroy and colleagues [28] to locate individuals within contexts that consist of diverse influences that affect an individual’s health seeking practices. This work aimed to focus as much attention on individuals as social and environmental factors, in terms of understanding how health problems arise and how health challenges can be overcome [28]. In HIV research in LMICs, socio-ecological models have been used in diverse international settings to understand how an individual’s health seeking practices are influenced by social, cultural, environmental, economic, political and legal factors at intra-personal, interpersonal and institutional levels of society [11, 12, 29, 30]. However, to our knowledge no studies have explored these issues with key populations in PNG or the broader Pacific. This paper examines the socio-ecological factors that encourage or hinder HIV treatment initiation and adherence among HIV positive key populations in PNG. Our findings could help inform service design and delivery, improve health outcomes of people with HIV, and reduce HIV transmission.

## Methods

A biobehavioural survey, known locally as *Kauntim mi tu* [Lit: Count me too] [17], was conducted among FSW and MSM/TGW were implemented in three sites in Papua New Guinea (Port Moresby, Lae and Mount Hagen) between June 2016 and December 2017. To complement the biobehavioural data, we conducted a qualitative sub-study involving semi-structured interviews among survey participants from each key population group in these three sites.

Participants were eligible to participate in the *Kauntim mi tu* if they were aged ≥12, able to speak English or *Tok Pisin*, and were able to provide informed consent. FSW had to be born female and sold/exchanged sex with a man in the past 6 months. MSM and TGW had to be born male and had oral/anal sex with another male-born person in the past 6 months. The low age criterion aligns with Papua New Guinea’s HIV and AIDS Protection and Management ACT [31] which states people ≥12 years do not require parental consent to participate in HIV testing / sexual health services/programs. *Kauntim mi tu* was a study designed to improve and provide sexual health services. Informed written consent was obtained from all participants and parental/guardian consent was waivered for those participants under the age of 18.

Study staff were trained in working with underage key populations and the need to ensure safety from harm and to identify and refer all sexually exploited persons, including all participants aged < 18 years who reported selling or exchanging sex, to partner organizations experienced in providing social and other protective services.

A purposive sampling method [32] was used to recruit 111 BBS participants into the qualitative sub-study. The 111 participants were purposively recruited [32] with the aim of reflecting the diversity of 2955 survey participants in terms of age, religious affiliation, place of origin, relationship status, HIV status, and sexual and gender identity. In order to ensure we reached a more diverse group of people, potential participants were not eligible to participate in the qualitative sub-study if they had previously participated in a recent qualitative study with the Papua New Guinea Institute of Medical Research. Participation in the qualitative study was voluntary and confidential. Participants were reimbursed 20 PNG Kina (US$6) for their time and transportation costs. The semi-structured survey was designed to address in detail the areas of the larger bio-behavioural survey from which they were recruited. These included, for example, sexual history and identity, condom use, family planning, stigma, social cohesion, violence, HIV knowledge, history of sexually transmissible infections, and uptake of health services. Interviews were conducted by Papua New Guinean researchers experienced in qualitative data collection techniques. Interviews lasted 40–60 min, were conducted in English or *Tok Pisin* and were digitally recorded. All interviews were transcribed verbatim and then translated into English, if required.

Data were input into NVivo 11 and coded thematically using deductive and inductive techniques [32] by one of the authors (MRM); this was followed by the first author (EM) performing further deductive analysis to examine individual (i.e. knowledge, attitudes, perceptions of risk), interpersonal (i.e. sexual partners, peers, family members) and institutional (i.e. health services) level influences on participants’ treatment initiation and adherence informed by a socio-ecological model of health promotion [28]. Inductive analysis was also conducted to identify specific issues within each theme.

This study was approved by the Papua New Guinea National Department of Health’s Medical Research Advisor Committee, the Research Advisory Committee of the National AIDS Council Secretariat, the Papua New Guinea Institute of Medical Research’s Institutional Review Board, the Human Research Ethics Committee at UNSW Sydney. The protocol was reviewed in accordance with the Centers for Disease Control and Prevention (CDC) human research protection procedures and was determined to be research, but CDC investigators did not interact with human subjects or have access to identifiable data or specimens for research purposes. To ensure confidentiality and anonymity, all names reported in this paper are pseudonyms.

This paper focuses specifically on interviews with participants who identified as HIV positive in the qualitative interview. Although TGW were included in both the survey and qualitative sub-study, no TGW identified as being HIV positive in the qualitative interviews. Therefore, the data used in this analysis refers only to HIV positive FSW and MSM (*N* = 28). In Port Moresby the number of HIV positive participants in the qualitative study was 8 while in Lae and Mount Hagen it was 13 and 7 respectively. Fourteen participants self-reported initiation of and adherence to ART, while 14 were not currently on treatment. These two groups (23 FSW and 5 MSM) are referred to as “taking ART” and “not taking ART” in the quotes presented in this paper (Table 1).

**Table 1:** Characteristics of study participants’

Participant characteristics	No. participants
*Key population group*	
- Female sex worker	23
- Men who have sex with men	5
*Recruitment location*	
- Port Moresby	8
- Lae	13
- Mount Hagen	7
*Location on cascade of care*	
- HIV+ taking ART	14
- HIV+ not taking ART	14
*Age group*	
- Under 19 years old	5
- 20–29 years old	12
- 30–39 years old	7
- 40–49 years old	3
- unknown	1

## Results

The findings of the study presented below are divided into individual, interpersonal and institutional factors that enhanced or inhibited effective treatment initiation and adherence among participants in the three study locations. While the behavioural and clinical data from *Kauntim mi tu* has been presented separately [18, 33], in this paper we draw on the qualitative data generated from both populations (FSW = 23; MSM =5) to examine the socio-ecological factors affecting key populations in PNG. We have successfully combined the qualitative data elsewhere to understand participation in the study, and the role that the provision of point-of-care testing played in participants willingness to participate [33].

### Individual influences

#### Influences enhancing health

For most participants on treatment, individual level factors motivated initial and continued engagement with HIV treatment services. The lived experience of continued good health influenced participants’ beliefs regarding the benefits of ART initiation and adherence.

I’ve taken my medicine correctly and look at me, I’m not in a wheelchair! I’ve taken ART for 6 years, and not once have I had to go to hospital and [be] put on a drip. Taking medicine is part of my life. Nothing of such happened in my life as I have continued up until now because of my adherence to medication … For this [ART] became part of my life so it really made me, like it placed a big impact on me … Why I will live is because of the adherence. (Bobby, MSM, aged 36 years, Lae, taking ART)

I started my treatment in 2005. A month later I saw changes in my physical appearance and my body came back to normal. I was in the sick bed for so long so I was so skinny and cannot move around but when I was put on ART it helps me to recover quickly … and the treatment also helps me to regain my weight … When I am adhering to my treatment then my viral load will always go down and my CD4 will rise. And I will have a positive mind so I will maintain my health and my body. (Fiona, FSW, aged 37 years, Mt Hagen, taking ART)

As indicated in the quote from Fiona, participants’ understanding of the biomedical aspects of treatment were evidenced by their explanations that ART suppressed viral load (‘go down’) and improved the body’s defence against HIV (‘CD4 will rise’). Although viral load testing was only made available for the first time in PNG during the *Kauntim mi tu* study [33], some participants understood the role of viral load testing in tracking the progression of HIV infection, demonstrated through their use of terminology such as undetectable when describing the results of viral load tests.

I also found out that my viral load is undetectable, and I realised my completed adherence to ART has given me this outcome. CD4 goes up and down but only viral load testing will tell you whether the ART is working. (Yano, FSW, aged 28 years, Port Moresby, taking ART)

When they gave me my result [viral load testing], I felt that I must continue to take my medication until my viral load is suppressed and bring my body’s defence up. (Carol, FSW, aged 20 years, Lae, taking ART)

#### Influences inhibiting health

Among HIV-positive participants not currently taking treatment, a disbelief of HIV test results, and difficulty with accepting their HIV status, inhibited linkage to care and treatment initiation. For some, this included a reluctance to commence treatment due to perceived good health and an absence of HIV-associated physical symptoms.

I went and got my blood tested, and the sister [nurse] she told me, “You have this kind of sickness [HIV], there are some small viruses moving around in your blood”. I never really thought that I had HIV. So [when] I heard it, I thought she was lying to me … I never took it seriously … From there I stayed [without treatment], and I no longer went back to them [clinic] … I didn’t feel any symptoms, I stayed normal so I was thinking, I have no diarrhoea, I have not felt body fever or back pain, or body been itchy; I didn’t feel it, and I thought I was alright … Why should I go and take this ART, I am going to drink this ART all my lifetime? I’m not positive, I’m negative, and I’m living healthy. (Sia, FSW, aged 40 years, Port Moresby, not taking ART)

Alcohol and drug (mis) use were noted to interfere with treatment adherence. In order to ensure they remain adherent, some participants disclosed they stopped consuming alcohol or drugs to ensure that they take their daily treatment at the designated time.

I’m still taking the medication [ART] but when I am drunk, I get muddled and I don’t take it. (Karu, FSW, aged 22 years, Lae, taking ART)

That time when I was still drinking and taking drugs, I’d usually forget [to take ART]. So I stopped drinking, and I quit marijuana and all that. I actually wanted to remain faithful to ART … I wanted to take my dose faithfully on time. (Pamela, FSW, aged 35 years, Mt Hagen, taking ART)

### Interpersonal influences – families, friends and peers

#### Influences enhancing health

For some participants, treatment initiation and adherence were motivated by interpersonal level factors, including their roles and responsibilities as parents (often mothers) and desire to have children in the future. The desire to prevent mother-to-child HIV transmission during pregnancy, childbirth and breastfeeding encouraged adherence and was shaped by a good understanding of the role of ART in preventing vertical HIV transmission. In the case of one HIV-positive MSM, this included supporting the mother of his child to also remain adherent to her treatment.

I usually take medicine [ART] every night [at] 8:00 pm. Because of the need to keep the adherence level and such at the same time, I encouraged the mother of my child to adhere to medicine too. If she doesn’t, it will be easy for her to infect the child through breastfeeding if you don’t adhere well to treatment. So I advised her to be very careful in this area. (Bobby, MSM, aged 36 years, Lae, taking ART)

I’m faithful with my medication [ART] and I know what this treatment does so I’m confident I’ll have a [HIV] negative baby. I will still be alive to take care of my children because I’m faithful to my medication. I know that medicine is prevention. (Yano, FSW, aged 28 years, Port Moresby, taking ART)

The desire to remain healthy to care for children and to watch children and grandchildren grow up also motivated participants to continue engagement in the HIV care cascade.

I will depend on this treatment [ART] until I see my grandchildren ... Both my parents are already old; therefore, I have to be strong by depending on ART only. Therefore, I never ignore my treatment. My foremost aim is to grow old with ART to see my grandchildren … I am positive so if I die then who is going to take care of my children? That is my main concern. (Pamela, FSW, aged 35 years, Mt Hagen, taking ART)

My children are my inspiration because I feel that if it wasn’t for ART, I wouldn’t be alive. The 11 years that I have been on treatment, I realise that it is because of ART, and I have [HIV] negative children and a [HIV] negative husband. These results have really motivated me to take ART every day, and so it’s almost impossible for me to forget to take it. I need to live to take care of my children and help them fulfill their future destiny. (Yano, FSW, aged 28 years, Port Moresby, taking ART)

Social support systems played an important role in enabling participants’ HIV care-seeking practices and ART adherence. For some FSW participants, family support facilitated ART initiation and adherence. This included caring for participants when they were sick, encouraging treatment initiation, accompanying them to medical appointments, providing financial support, and concealing HIV status to avoid HIV-related stigma.

He [my brother] took me from where I was living to go and live in his house where he took care of me … There were no women to look after me, just my brother I was in a very bad condition. I was wearing diaper and was in a coma, I couldn’t talk … So through the love, concern, support and care from my brother I did not die, which I was supposed to. But I live with him for so long until I started my treatment in 2006. My brother brought me to Mount Hagen hospital, and we were confused of where we are going to access the services as I’m the first person in the family to contract the HIV virus. So, from here we were referred to the clinic, and there I started my treatment from then onwards. (Fiona, FSW, aged 37 years, Mt Hagen, taking ART)

Only my mother and my stepfather know. … that I have this infection [HIV] … I always lie to them [other relatives] that I have TB, and I usually tell them that I am on TB treatment and not on this virus treatment [ART] … [If they found out] I would have to leave that place and go live somewhere and die there. (Carol, FSW, aged 20 years, Lae, taking ART)

Friends and peers supported a few participants by sharing information regarding ART adherence and by providing guidance on managing medication side effects.

I have not told anyone [about HIV status]; it’s personal, and I don’t tell my family … One of my best friends also has this disease so both of us only know … when we go to get the supply [ART], we use to share what the doctor said, they told me such and such, we must take it like such, one or two days we are not going to skip it. Every night we must take it, both of us we used to plan like that. (Baria, MSM, aged 33 years, Port Moresby, taking ART)

My two girlfriends … only know [about HIV status] so they used to take me to the hospital and such. My first time to take the HIV medicine, it was very strong, and I didn’t know … When I see people, I was a drunkard woman walking amongst them, and when I wanted to talk, I thought that I was talking sense, but my words went confused. Then my girlfriend said, “I know that you took the medicine … that is why you are confused, so you need to sleep. After you have taken it for some three or four month, such then you would get used to taking it. This is your first time, so you are going to get dizzy.” (Karu, FSW, aged 22 years, Lae, taking ART)

Some participants adhered to ART while keeping their HIV-positive status and treatment regimen secret from family and friends to reduce the likelihood of experiencing HIV-related stigma and discrimination. Most notably, participants used concealment to prevent unwanted disclosure, such as taking medication discreetly to avoid being seen, saying that medication is for another health condition when asked, and transferring medication into different containers.

They [family] ask why I keep the medicine [ART] in the drawer. I used to tell them not to touch it … I never take my [ART] medicine right in their presence. However, I take it away from them. (Kawas, MSM, aged 21 years, Lae, taking ART)

She [daughter] usually says, “Mum, you are not sick so why do you take drugs all the time?” and I usually say, “oh, I have a problem in my stomach.” That’s what I say when I take my ART. (Yano, FSW, aged 28 years, Port Moresby, taking ART)

Participants also believed that ART helped conceal their HIV-positive status from others through reducing HIV-related physical symptoms (e.g., weight loss, poor health, skin issues), which encouraged ART adherence.

When I take ART, I am normal, I never get sick. I look healthy, and when people see me, they can’t tell that I’m sick [have HIV]. (Karu, FSW, aged 22 years, Lae, taking ART)

People usually think that I’m a good woman, HIV negative. My family doesn’t even know about my status. Only my workmates know about my status … When I went on treatment my appearance came back to normal again. (Pamela, FSW, aged 35 years, Mt Hagen, taking ART)

#### Influences inhibiting health

Family members, including partners and parents were noted as inhibiting a few participants ART adherence. This included one FSW whose access to treatment was impeded due to fear that an abusive ex-partner may track her down at the HIV clinic and another whose parents discarded her supply of medication on learning of her HIV positive status.

When I left it [stopped taking ART], that was when I left the father of the children and ran away. It must have been 5 months that I left medicine and went off … I was hiding around, and I said, he might know the clinic which I usually get the medicine. So I might go there, and he might come and do some things [physical violence] to me, so I won’t go and get it. (Maria, FSW, aged 25 years, Port Moresby, taking ART)

For 2 months I have not got the medicine [ART] … 2 months I haven’t taken it [because] my mother found out and burned the medicine. She called my father, my stepfather, then they took my medicine and threw it away. I cannot go back and get treatment because my health book is in the village. (Ato, FSW, aged 30 years, Lae, not taking ART)

At the time of our study, and still mostly the case in government services, HIV treatment in PNG is provided in stand-alone clinics or integrated into a sexual health clinic. Therefore, the risk of being seen while accessing HIV treatment, and consequently subjected to shame, discrimination and rejection from family and community acted as a disincentive to some participants accessing HIV care.

They [health worker] told me to come back [to get ART] but I didn’t … I gave up, and I didn’t go back, I was shy to go … [I was worried] people who know me would see me, and they would go around gossiping … and say, “heeii that woman usually goes and get AIDS medicine.” I was ashamed so was staying up until now [without ART]. (Amber, FSW, aged 24 years, Mt Hagen, not taking ART)

### Institutional influences – within health services

#### Influences enhancing health

At an institutional level within health service settings, supportive patient-provider relationships positively influenced participants’ experiences within HIV care services. The support and guidance of healthcare workers to navigate HIV services after diagnosis and reassurance of the ability to live a long and healthy life with HIV facilitated participants’ treatment initiation and subsequent adherence.

When I went in, she [nurse] said, “You have HIV, you have the virus.” I started to cry … The nurse advised me not to cry. She said, “It’s not like it’s AIDS; HIV does have medication, so we can help you and refer you to the hospital, and you will receive medication from there. (Carol, FSW, aged 20 years, Lae, taking ART)

The doctor gave me some advice and made my head spin inside. I told him that I won’t take the treatment [ART], and I said, “Let me die away, and let me be,” but he said, “No, you are a very young 18-year-old girl, and you have a very long way to go” … He explained that those who depended on this treatment can live to grow grey hairs with it, so don’t worry … He gave me very tough advice … so I came back here in the morning the next day … [and] I was given treatment [ART]. (Julie, FSW, aged 18 years, Mt Hagen, taking ART)

#### Influences inhibiting health

While a supportive patient-provider relationship within HIV care services positively influenced some participants’ HIV care and ART adherence, others spoke of negative experiences with healthcare workers, experiences that inhibited sustained engagement in the HIV care cascade. Some participants stated that healthcare workers scolded them for non-adherence to ART and/or spoke disparagingly toward them.

[A] couple of times I didn’t take the medicine [ART], they [healthcare worker] became really angry and got cross with me saying that I could be another dead person; they told me I cannot skip my medicine. (Mofa, FSW, aged 20 years, Lae, not taking ART)

Sometimes I don’t feel like going … they don’t talk to me kindly. What they [health worker] said was, “If you want to die then go and just die. Do you pay us to work here?” That’s what they say … She even talked about the system and all that, and she really made us [FSW] looked like fools, and that’s what I don’t like … So we left, and we left for good. (Neeta, FSW, aged 24 years, Mt Hagen, not taking ART)

The lack of anonymity in health centres where HIV care is delivered, as evident for example, by the large HIV and voluntary counselling and testing signs outside, also inhibited ART adherence. Negative interactions with and experiences of HIV-related stigma and discrimination by others at the hospital and clinics also discouraged ART adherence.

When we go, many times like people usually look at us and gossip, they would come, some would come for their TB drugs or come to check their blood … many of them would come stand, pretend to stand and stare … Some [patients] would come and say, “That’s it, they have been doing it, and they got infected, and now they came to get treatment.” (Ato, FSW, aged 30 years, Lae, not taking ART)

## Discussion

In a national context where sex work and male-to-male sex are illegal and highly stigmatised and HIV prevalence is concentrated among key populations, our findings demonstrate a range of socio-ecological influences that can either encourage or inhibit HIV treatment initiation and adherence among these populations in PNG. Although several qualitative studies have examined living with HIV, including treatment beliefs and experiences, in PNG, to our knowledge, our analysis is the first to solely focus on and explore the experiences of key populations relating to ART and adherence in this setting. As such, our findings contribute to the growing body of literature in the South and South East Asia region that seeks to understand the drivers and barriers that influence key populations’ engagement across the HIV care cascade [11, 12, 22–27]. Our socio-ecological [28] analysis provides a holistic understanding of the diverse factors influencing ART initiation and adherence among key populations in the three largest cities, including the national capital, in PNG.

We found that individual, interpersonal and institutional factors encouraged ART initiation and adherence among participants across the three study sites. Similar to other key populations in the region [12, 14, 25, 27] and other LMICs, [10, 34] an understanding of the biomedical aspects of treatment – increased CD4 and reduced viral load – and experiences of the social and health benefits of treatment adherence influenced participants’ initial and continued engagement in HIV care. FSW demonstrated greater understanding of HIV treatment aspects, particularly viral load testing compared to MSM. More HIV positive FSW were interviewed than there were HIV positive MSM, and this may have affected this. However, it is more likely that the MSM interviewed were less connected to peers and networks of key populations and their civil society organisations. Generally speaking, in PNG FSW are better connected than MSM and act as peer educators and therefore more aware of HIV and the tests used to understand and measure clinical performance. MSM are more diverse as a group, often married and have sex with other men in secret. This was in part why we sought to recruit more widely and prioritised qualitative interviews with those who had not previously done so.

Not surprisingly the desire to remain healthy to care for children or have children in the future facilitated treatment initiation and adherence [11, 22, 23, 35], particularly for FSW. An understanding of the role of treatment in reducing the risk of vertical HIV transmission in future pregnancies encouraged treatment adherence among some participants, including MSM. For some FSW, social, emotional and financial support provided by family and friends enabled access and adherence to treatment and included such things as caring from HIV positive people when they were sick, accompanied treatment visits, and concealing HIV status from others. For MSM, social support from peers assisted with HIV treatment and ART adherence and some disclosed keeping their HIV diagnosis and treatment regime secret from family in order to reduce the likelihood of HIV-related stigma and remain adherent to ART. It is also likely that keeping their HIV status a secret also prevented any unwanted disclosure of their sexuality, or at least risk category. Passing as HIV negative was probably not as important for some as it was passing as cis-gender. Similar findings have been noted in other studies in PNG [36, 37] and among key populations in LMICs [27, 34]. Friendly, non-judgmental and supportive patient-provider relationships that assisted participants navigate HIV services after diagnosis and ensured early ART initiation enabled HIV care [11, 12, 24, 27, 34]. Different experiences of intersecting socio-ecological influences made accessing HIV treatment and remaining adherent to ART less challenging for some participants. For example, those with a good understanding of the health benefits of ART, strong social support from family and peers, and supportive and safe service provision were more likely to report continued engagement in the HIV care cascade.

Conversely, a range of socio-ecological influences inhibited treatment initiation and adherence. Complementing findings from research with other key populations in LMICs [14, 26], we found that perceptions of personal good health and a refusal to accept one’s HIV-positive diagnosis were barriers to ART initiation. Alcohol and drug use impaired adherence among some FSW [10, 25, 38]. Lack of family support after the HIV-positive diagnosis and fear of shame, gossip and even physical violence if seen in the location of HIV services prevented some FSW from accessing HIV services. Negative interactions with healthcare workers, lack of anonymity within HIV services, and perceived or actual experiences of HIV-related stigma and discrimination inhibited participant’s willingness to remain engaged in HIV care [11, 24–27, 39]. It was the absence of these barriers that participants in the qualitative study identified as important in influencing their participation in the larger biobehavioural survey – *Kauntim mi tu* [33]. When participants experienced barriers at multiple socio-ecological levels (e.g., stigma and discrimination from family, peers and health workers due to HIV positive status and/or sexuality/job) HIV treatment uptake and adherence was particularly challenging.

### Limitations

Our study had several limitations. This qualitative study was designed to understand the issues of key populations in relation to several domains including sexuality, condoms, violence and STI and HIV prevention, treatment and care, but it was not designed to specifically understand the experiences of HIV-positive key populations and their relationship to the HIV care cascade. No TGW participants in the qualitative sub-study identified as being HIV positive so we are not certain that the issues faced by FSW and MSM can speak to all the issues faced by TGW, particularly for example the unique issues faced by TGW in relation to gender affirming HIV care and support. Based on the burden of HIV among key populations, and as PNG expands HIV viral load testing, and as issues with HIV drug resistance and adherence continue, it would be helpful to examine engagement with the HIV cascade among key populations in more detail.

### Policy and program implications

The HIV response in PNG includes efforts to engage key populations, including FSW, MSM and TGW, in all stages of the HIV care cascade, [20] but challenges remain to reach the UNAIDS 95–95-95 targets among these population groups. While we do not assert that these same issues do not reflect those of others living with HIV in PNG, several areas for action to increase HIV treatment uptake and adherence for key populations were identified.

First, our findings demonstrate the need for health promotion messaging centred on the positive health impacts of early treatment initiation and ART adherence. Drawing on data from the individual and interpersonal dimensions of the socio-ecological model, this could include messaging that acknowledges the desire of people living with HIV to be devoted parents, partners and family members and remain healthy to care and provide for family [11, 40]. Health promotion messaging that aims to reduce stigma throughout communities and health services would also help to build a safe environment for key populations in PNG. Further research could identify how best to tap into interpersonal relationship to communicate health promotion messages to key populations in PNG.

Second, changes at the institutional levels within health care services are required. For instance, anonymous, flexible, client-friendly HIV services tailored to the unique needs of key populations in PNG could improve ART initiation and adherence, as highlighted elsewhere [33]. Providing support and training in these areas for existing and new health service staff could help prevent poor service experiences, avoid the chance of stigmatising interactions between service providers and clients, and improve the interpersonal relationships between clients and service users. Another lesson from the interpersonal analysis points to the benefits of using peer support groups and leadership roles for key populations within HIV services could further strengthen service delivery and increase key populations’ engagement in HIV services. Scaling up point-of-care HIV viral load testing across the country could help guide monitoring and provide opportunities for targeted motivational adherence counselling, which could reduce disease progression and transmission in PNG [4, 5].

Third, drawing on experiences from high HIV prevalence countries [41–44] and recent calls from the World Health Organization for a shift in HIV service delivery for key populations from clinical settings into community settings, [8, 45], piloting HIV testing, care and ART distribution in community-based settings (not health clinics) could facilitate treatment initiation and increase ART adherence among key populations in PNG, complementing existing recommendations for community-based HIV testing and ART initiation at time of HIV diagnosis for key populations [46]. Treatment in community-based settings aligns with global calls for a more ‘people-centred approach’ to HIV testing and treatment [47] that reduces structural barriers often experienced by key populations [44].

## Conclusion

Ensuring widespread access to ART initiation and adherence will address the high burden of HIV and low treatment coverage among all key populations in PNG. Our findings offer qualitative insights that can help HIV programs think about how to ensure that HIV services respond to the needs and concerns of key populations living with HIV in PNG. Exploring health promotion messages that highlight the positive health impacts of early ART initiation and adherence; providing client-friendly services and community-based treatment initiation and supply; and providing HIV viral load testing across PNG could increase ART initiation and adherence and improve health outcomes for key populations in PNG.

## Data Availability

The datasets generated and analysed during the study are not publicly available on a repository as this goes against the Institutional Review Board approval of the Papua New Guinea National Department of Health’s Medical Research Advisor Committee, the Research Advisory Committee of the National AIDS Council Secretariat, the Papua New Guinea Institute of Medical Research’s Institutional Review Board, the Human Research Ethics Committee at UNSW Sydney. Relevant excerpts of the data are in the manuscript. For researchers who meet the criteria for access to confidential data, their request will be evaluated on a case-by case basis. Data inquiries may be directed to Associate Professor Angela Kelly-Hanku, a.kelly@unsw.edu.au

## Abbreviations

ART: Antiretroviral therapy
CDC: Centers for Disease Control and Prevention
FSW: Female sex worker
HIV: Human immunodeficiency virus
LMIC: Low- and middle- income countries
MSM: Men who have sex with men
PNG: Papua New Guinea
TGW: Transgender women
UNAIDS: Joint United Nations Programme on AIDS
